# X‐Ray Multibeam Ptychography at up to 20 keV: Nano‐Lithography Enhances X‐Ray Nano‐Imaging

**DOI:** 10.1002/advs.202310075

**Published:** 2024-06-23

**Authors:** Tang Li, Maik Kahnt, Thomas L. Sheppard, Runqing Yang, Ken V. Falch, Roman Zvagelsky, Pablo Villanueva‐Perez, Martin Wegener, Mikhail Lyubomirskiy

**Affiliations:** ^1^ Centre for X‐ray and Nano Science CXNS Deutsches Elektronen‐Synchrotron DESY Notkestr. 85 22607 Hamburg Germany; ^2^ MAX IV Laboratory Lund University Box 118 Lund 221 00 Sweden; ^3^ Karlsruhe Institute of Technology Institute for Chemical Technology and Polymer Chemistry Engesserstr. 20 76131 Karlsruhe Germany; ^4^ Leipzig University Institute of Chemical Technology Linnéstr. 3 04103 Leipzig Germany; ^5^ Division of Synchrotron Radiation Research and NanoLund Department of Physics Lund University Lund 22100 Sweden; ^6^ Karlsruher Institut für Technologie Institut für Angewandte Physik Wolfgang‐Gaede‐Straße 1 D‐76131 Karlsruhe Germany

**Keywords:** lens‐less imaging, microscopy, nano‐lithography, ptychography

## Abstract

Hard X‐rays are needed for non‐destructive nano‐imaging of solid matter. Synchrotron radiation facilities (SRF) provide the highest‐quality images with single‐digit nm resolution using advanced techniques such as X‐ray ptychography. However, the resolution or field of view is ultimately constrained by the available coherent flux. To address this, the beam's incoherent fraction can be exploited using multiple parallel beams in an X‐ray multibeam ptychography (MBP) approach. This expands the domain of X‐ray ptychography to larger samples or more rapid measurements. Both qualities favor the study of complex composite or functional samples, such as catalysts, energy materials, or electronic devices. The challenge of performing ptychography at high energy and with many parallel beams must be overcome to extract the full advantages for extended samples while minimizing beam attenuation. Here, that challenge is overcome by creating a lens array using cutting‐edge laser printing technology and applying it to perform scanning with MBP with up to 12 beams and at photon energies of 13 and 20 keV. This exceeds the measurement limits of conventional hard X‐ray ptychography without compromising image quality for various samples: Siemens star test pattern, Ni/Al_2_O_3_ catalyst, microchip, and gold nano‐crystal clusters.

## Graphical abstract

Hard X‐ray multibeam ptychography paves the path toward high‐resolution, non‐destructive 3D imaging of mm‐sized samples. The present study describes the achievement of imaging of 0.1mm+ samples using up to 12 parallel uniquely coded X‐ray beams with energies of up to 20 keV. Cutting‐edge laser technology enables 3D printing of a high aspect ratio lens array for hard X‐ray parallel focusing.

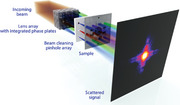

## 1 Introduction

Microscopy is a core driving force in science and technology. It plays a pivotal role in understanding the structure and function of materials by delivering access to micro‐ and nanoscale information. To date, sub‐µm resolution is routinely achieved with visible light microscopy. In contrast, using electron microscopy, it is possible to exceed single‐digit  nm resolution. Unfortunately, these methods are either constrained to retrieve only surface information (e.g., scanning electron microscopy (SEM)) of large samples, involving invasive subsampling of  nm‐thin samples (e.g., transmission electron microscope (TEM)), or  nm‐sized volumes for TEM tomography, or total sample destruction (e.g., FIB‐SEM). Compared to the above methods, the high penetration power of hard X‐rays enables non‐destructive studies of relatively large samples. Among other benefits, this enables a more representative investigation of complex or hierarchically structured samples while minimizing invasive subsampling. This is particularly relevant in the study of functional materials such as catalysts,^[^ ^1^ ^]^ energy materials,^[^ ^2^, ^3^ ^]^ or nanostructured devices such as microchips for example. Such samples are typically complex composite structures in which small sub‐volumes may not be representative of the parent object. In this context, X‐ray microscopy (XRM) and computed tomography (CT) at third‐generation synchrotron radiation facilities (SRFs) have experienced explosive development in the last three decades.

Currently, XRM is an indispensable and flexible tool for studying extended samples (e.g., nm to cm scale) with wide resolution ranges reaching up to single‐digit nm. The highest resolution measurements have been enabled by the development of lens‐less imaging methods such as coherent diffraction imaging (CDI)^[^ ^4^ ^]^ and ptychography.^[^ ^5^, ^6^, ^7^, ^8^, ^9^ ^]^ In hard X‐ray ptychography, the sample is irradiated by a confined coherent beam, with the diffraction signal recorded by a detector. By measuring the beam position on the sample and recording the diffraction signal, the sample complex transmission function and the probing beam are iteratively reconstructed. As a lens‐less imaging technique, ptychography exploits focusing optics purely to increase photon fluence on the sample and speed up data collection. Another aspect of ptychography is that the incoming beam needs to be fully or nearly fully coherent. Since the typical coherent fraction of the beam at modern third‐generation SRFs is less than 1 %, this means that nearly 99% of the incoming beam is wasted. Notably, ptychographic measurements are also possible with electron microscopy where they operate at an even higher resolution than conventional TEM, although they are still limited to very small sample volumes.^[^ ^10^, ^11^ ^]^

The major shortcoming of ptychography is that resolution is ultimately limited by the X‐ray beam flux at the sample. Consequently, assuming a certain flux and finite beamtime allocation at a given SRF, either the sample size or target resolution must be constrained. This hinders the core advantage of XRM (i.e., high‐resolution imaging of extended samples) in either of two ways. First, by limiting the sample size in order to limit measurement duration. This can force invasive subsampling and potentially unrepresentative imaging of complex samples. Second, by compromising the measurement resolution in order to increase sample size, therefore rendering many in situ studies of transient processes unfeasible due to lack of sensitivity to small changes in the sample. While beamtime availability therefore restricts the measurement of extended samples at maximum resolution, an additional challenge comes from X‐ray attenuation. As sample thickness increases, higher photon energies are needed to penetrate thicker samples. High energy ptychography is therefore an attractive prospect, due to minimal attenuation and coincidentally decreased beam damage due to smaller absorption cross‐sections. However, considering that the available coherent fraction of photons at SRFs is inversely proportional to energy, high‐energy ptychography is challenging in practice due to insufficient coherent flux. For this reason, only a few studies have reported on X‐ray ptychography at energies above 17 keV.^[^ ^12^ ^]^

Resolving the issues above would enable high‐resolution imaging of extended samples on feasible timescales. However, performing ptychography under these conditions constitutes one of the most challenging issues in modern X‐ray imaging. One approach to resolve this is with fourth‐generation diffraction‐limited SRFs offering increased coherent flux in the hard X‐ray regime. However, such facilities are prohibitively expensive and time‐consuming to construct. The central issue of limited beamtime availability cannot feasibly be resolved by simply building more SRFs. An alternative approach developed in recent years involves the inclusion of previously unusable photons into ptychographic experiments and reconstruction algorithms. This approach is called multibeam ptychography (MBP), and is based on dividing an incident X‐ray beam into multiple beams which are in themselves coherent, but mutually incoherent. Thus, the requirement for beam coherence for each individual focused beam is the same as for conventional single‐beam ptychography: the single optical element aperture needs to be coherently illuminated. MBP was first demonstrated in 2017 with visible light,^[^ ^13^ ^]^ and later with X‐rays.^[^ ^14^, ^15^, ^16^, ^17^ ^]^ Since multiple regions of the sample are imaged simultaneously, this speeds up ptychography on a linear scale with an increasing number of beams. This is equivalent to performing multiple conventional ptychography measurements simultaneously. Development of MBP is therefore of great interest in the context of high‐resolution X‐ray imaging on larger and more representative samples, or with rapid acquisition rates, such as in the study of functional materials, catalysis, or nanofabrication.

The major challenge in X‐ray MBP involves using a larger fraction of the incoming beam at higher energies where gains in measurement speed and sample size are the most relevant. Various types of X‐ray optics have been proposed for this purpose, such as an array of Fresnel zone plates (FZPs),^[^ ^14^ ^]^ focusing mirrors with slits,^[^ ^15^ ^]^ and compound refractive lenses (CRLs).^[^ ^16^, ^17^ ^]^ The criteria for efficient MBP with hard X‐rays are: 1) ptychography requirements need to be met, e.g., confined and coherent illumination, and 2) optics need to be highly efficient. Since no off‐the‐shelf optics exist for MBP, custom solutions are needed. While FZP has well‐established manufacturing processes, they are unsuitable due to their low efficiency (below 10%) at X‐ray energies above 12 keV.^[^ ^18^ ^]^ Mirrors are only usable in combination with other optics (e.g., FZP, slits) due to mechanical constraints, which ultimately hinders the photon efficiency and practicability of such schemes. The proven most feasible option is therefore double‐concave CRLs.^[^ ^19^, ^20^, ^21^, ^22^ ^]^ Another challenge in MBP is that the beams must be sufficiently unique in phase and/or amplitude in order to achieve robust separation of overlapping signals from multiple beams at the detector. It has been shown that specifically designed different phase plates^[^ ^23^ ^]^ added to each lens stack can achieve this.^[^ ^17^ ^]^

The pivoting point in the development track of MBP was the application of enabling technology—3D laser two‐photon absorption printing technique^[^ ^24^ ^]^ for manufacturing focusing optic arrays was used to create focusing elements out of polymer with full geometric freedom, high precision, and costs comparable to and even less than those for manufacturing FZPs. Then, the highest known MBP photon utilization of 98% was achieved at energies of 7 and 9 keV with double‐concave CRLs in tightly packed arrays.^[^ ^17^, ^22^ ^]^ A radius of curvature of a single parabolic surface of single‐digit µm was achieved. To perform MBP at 20 keV (compared to previous measurements performed at 6.5–9 keV), the focusing power of a single lens tower requires an order of magnitude smaller effective lens curvature (effective curvature = single lens curvature/number of lenses), from state‐of‐the‐art 0.83 µm^[^ ^17^ ^]^ down to 100 nm level as the lens focusing power decreases quadratically with the X‐ray photon energy (focal length is linearly proportional to the real part of the complex refractive index^[^ ^25^ ^]^). If this is not achieved then the flux density of the non‐scattered beam at the detector will be increased (due to the smaller beam size) which will cause earlier pixel saturation. The low optical density of polymers is therefore a major weakness of laser‐printed optics in the hard X‐ray regime. On the other hand, no alternative manufacturing process can currently deliver tightly packed arrays of refractive lenses. To achieve large focusing power multiple individual lens elements have to be stacked together, leading to an increased lens tower aspect ratio and consequently more strict requirements for manufacturing precision. This sets very challenging constraints on manufacturing as the lens tower aspect ratio reaches 100:1, keeping in mind that manufacturing precision is crucial to the overall success of the MBP measurements.

Unlike previous experiments with MPB, the most challenging of which were constrained to a maximum of three parallel beams^[^ ^17^ ^]^ and lower X‐ray energies (6.5,^[^ ^15^ ^]^ 7,^[^ ^16^ ^]^ 8,^[^ ^26^ ^]^ 8.8,^[^ ^14^ ^]^ and 9 keV^[^ ^17^ ^]^) here we report on the first application of MBP with up to 12 beams at irradiation energies of 13 and 20 keV. This was accomplished by developing a lens array using cutting‐edge laser printing technology. The quality and robustness of MBP are demonstrated on a range of sample systems representing diverse applications, including traditional test patterns, a microchip, porous catalyst structures, and gold nano‐crystal particles. Hard X‐ray MBP is therefore demonstrated as a method of the highest potential to achieve extended imaging of large samples at the nanoscale, which is otherwise temporally unfeasible even with state‐of‐the‐art single‐beam X‐ray ptychography.

## 2 Results

**Figure 1:**
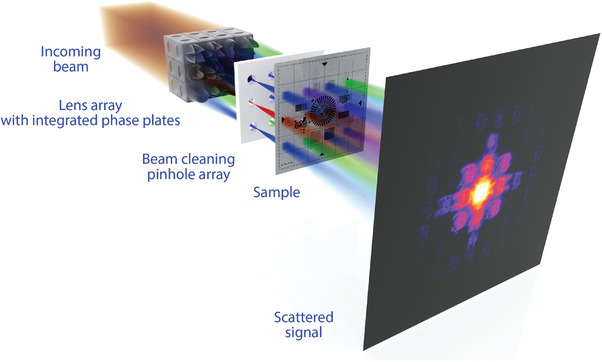
Scheme of the performed multibeam ptychography showing separation of the primary beam into individual coded beams which simultaneously irradiate multiple sample points. Scattered beams are propagated to the detector analogously to conventional hard X‐ray ptychography. Typical distances are: lens array to sample—10 cm, pinhole array to sample—2 cm, sample to detector—400 cm.

The general measurement scheme for MBP is depicted in **Figure** **1**. Experiments were performed at two beamlines: P06 at PETRA III (13 keV) and ID13 at ESRF‐EBS (20 keV). The experiment at the P06 beamline was performed at the mechanically stable instrument PtyNAMi: ptychographic nano‐analytical microscope.^[^ ^27^ ^]^ The total desired number of beams was selected using slits upstream (not shown in the scheme) of the lens array. For all beams combinations, the scan range was 35 × 35 µm to cover the distance between neighboring beams of 30 µm with 5 µm of overlap of sample regions scanned by adjacent beams.

### 2.1 Lens Arrays Design, Manufacturing, and Corrections

**Figure 2:**
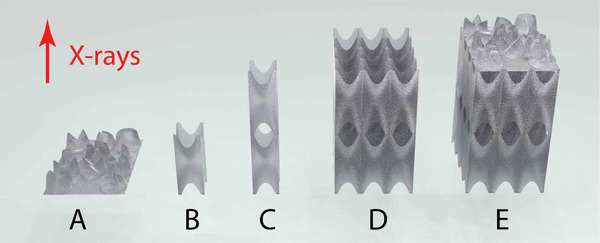
Examples of optical elements lens arrays are comprised from. A—phase plates arranged in 3 × 4 array. B—double‐concave lens. C—lens tower comprised of two double‐concave lenses. D—lens array comprised two double‐concave lenses along the beam and 3 × 4 across the beam. E—lens array with phase plates on top.

Lens arrays (**Figure** **2**D) designed in this work have two key elements: lens tower (Figure 2C), comprised of different numbers of individual double‐concave lenses (Figure 2B) arranged along the beam and phase plates, located at the end of each tower (Figure 2A). Both lens arrays designed for different energies had lens towers arranged in a 3 × 4 grid across the beam. The lens array designed for experiments at 13 keV contained 30 double‐concave lenses along the beam, and another one containing 40 double‐concave lenses was designed for experiments at 20 keV. For simplicity, we will call them 30‐lens array and 40‐lens array.

The lens arrays were manufactured and subsequently corrected, with experimental validation in between. For this purpose, optics performance was assessed with 13 keV irradiation at the synchrotron. Each lens tower in the array was individually characterized with single beam ptychography, and the reconstructed complex wave fields were propagated numerically. After the tests, it was discovered that in the first iteration, the two central towers (from 3 × 4 grid) had different focal lengths with respect to the border towers, possibly due to the dose accumulation during laser printing. This led to a size mismatch between the two central beams and the remaining beams. This directly affected the efficiency of MBP scans, since the scan step size was reduced and measurement time therefore increased due to overhead to each step. According to sampling requirements, the overlap of the irradiated sample regions in adjacent scan positions should exceed 60%. The experimental tests of the corrected lens towers (second iteration) at 20 keV showed the expected joint lens towers' performance, providing sufficient experimental conditions for executing MBP with different beam arrangements.

**Figure 3:**
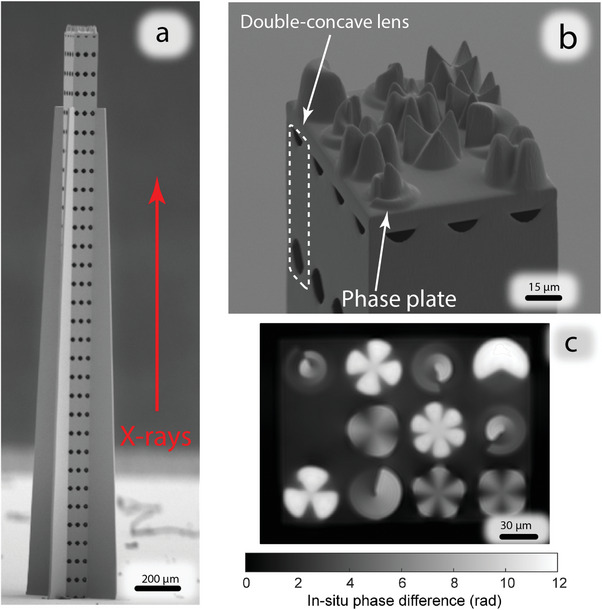
a) SEM image of a 30‐lens array in a 3 × 4 grid for 13 keV photon energy with phase plate on top. b) SEM close‐up image of phase plates and individual double‐concave lens. c) In situ QPI of a separately printed phase plate showing phase difference between unpolymerized and polymerized photoresist. The phase was measured with a wavelength of 630 nm.

**Figure** **3**a shows an example of a printed lens tower array for 13 keV photon energy. A close‐up of the phase plate on top of the lens system is shown in Figure 3b. The quality of integrated phase plates on top of each lens tower is especially important since the separation of the signals from different beams relies on differences in their phase and/or amplitude. For this purpose, we characterized the phase plate using a recently developed in situ quantitative phase imaging (QPI) technique.^[^ ^28^ ^]^ A resulting in situ phase topography map measured on a separately printed phase plate is depicted in Figure 3c.

### 2.2 Multibeam Ptychography at 13 keV: Six and 12 Beams

**Figure 4:**
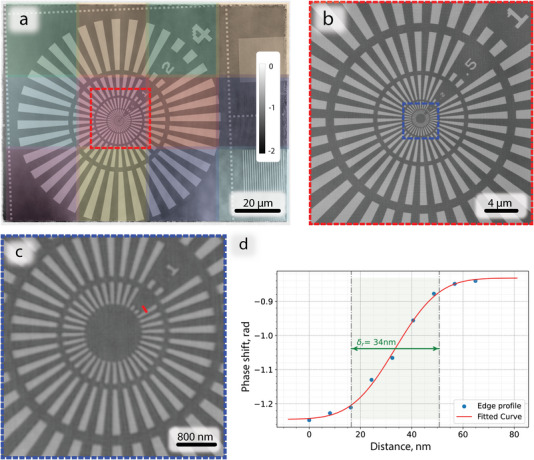
Reconstructed object phase from the 12‐beam measurement of the Siemens star test pattern at 13 keV; a) overall image, colored squares represent regions irradiated by each individual beam respectively; b) magnified central region partially scanned by two neighboring probes; c) magnified region with smallest features—50 nm, red line indicates the position for the line profile; d) line profile of the edge estimating resolution; The color bar represents phase shift in radians. The pixel size in reconstruction is 8 nm.

A series of samples with different structural features and/or complexity were used to validate the performance of the MBP measurements and MBP reconstruction algorithms. First, a Siemens star XRESO‐50HC^[^ ^29^ ^]^ manufactured by NTT‐AT with the smallest features of 50 nm was imaged. For the 12 beam arrangement a full lens array was illuminated. This resulted in an effective scanned area of 95 µm × 125 µm. The reconstructed phase image of the Siemens star is depicted in **Figure** **4**, with color shading used to indicate the 12 separate regions of the sample irradiated by each of the 12 probes respectively. This color indication made it easy to inspect regions where signals from different probes needed to be “stitched” by the reconstruction algorithm. This information was used to check for possible artefacts or errors in the reconstruction process. For example, the magnified central area was scanned with two individual probes (Figure 4b), whereby no visible artifacts are present in the reconstruction, and the 50 nm bars are clearly resolved Figure 4c. This indicates that the diffraction signals from different sample parts were robustly deconvoluted by the reconstruction algorithm, providing high‐quality reconstructions. Moreover, the line edge profile (Figure 4d) indicates a resolution of 34 nm (edge response), which is comparable with the result of a single beam reconstruction of 34 nm from the scan taken for the characterization of single probes with normalized statistics per irradiating beam—see comparison in Supporting Information I. In summary, MBP is therefore directly comparable in terms of resolution with conventional single beam ptychography,^[^ ^17^ ^]^ while facilitating rapid scans over large fields of view through simultaneous measurement of multiple sample positions.

**Figure 5:**
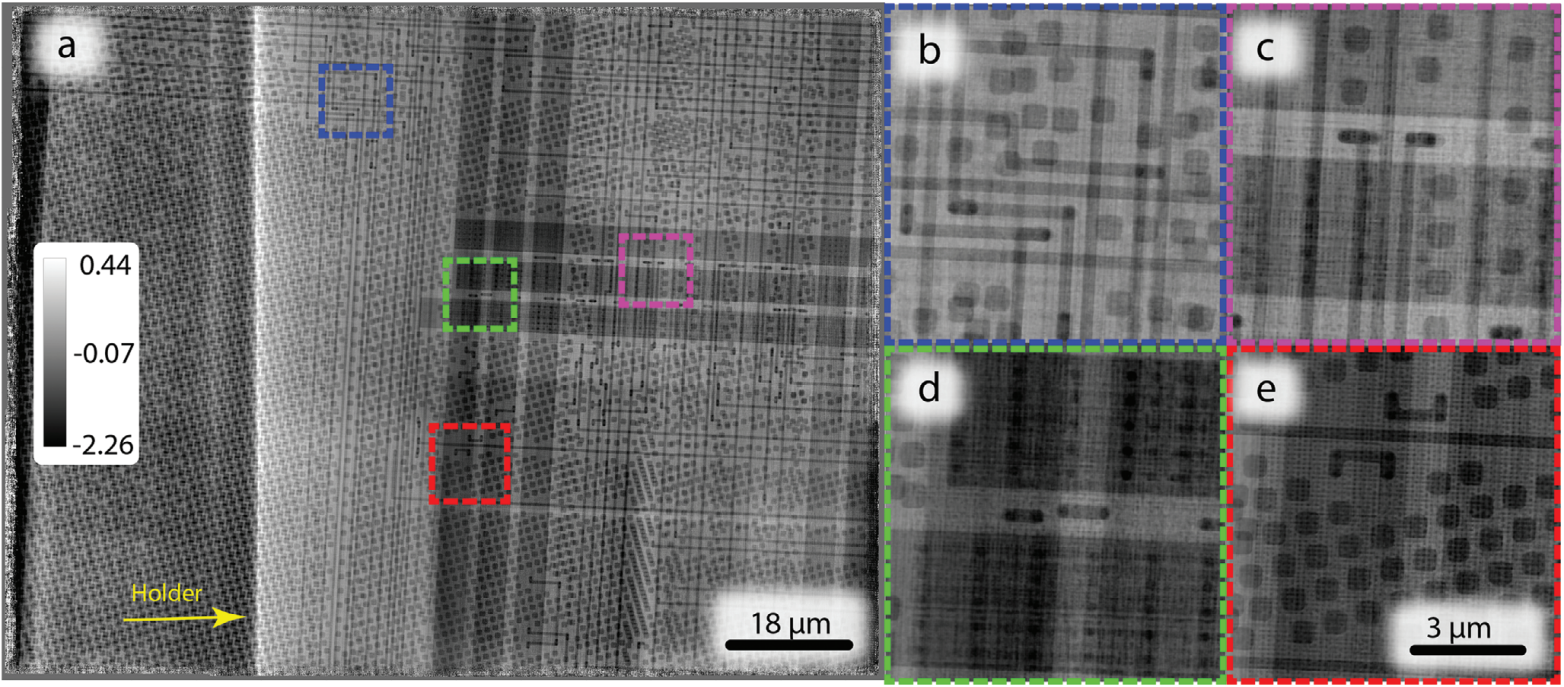
Reconstructed object phase from the 12 beam measurement of the microchip at 13 keV; a) overall image; b–e) magnified regions indicating small features; The color bar represents phase shift in radians. The pixel size in reconstruction is 16 nm.

To further assess the capabilities of MBP, samples with more diverse features were examined. These represent real objects that form the basis of potential application areas for MBP. First, a microchip containing heterogeneous pattern structures of circuits, transistors, and contacts spread over several layers was scanned with 12 beams in the same manner as described above. As an extended planar object, the microchip represents a potential application that ideally performed by scanning an array of beams in MBP instead of just one beam in conventional single beam ptychography. The reconstructed microchip is depicted in **Figure** **5**. The sample visibly contains a large number of different scale features, from several µm – a “trench” originating from the top of the image (Figure 5a) to tens of nm ‐ dots (Figure 5e). All of these features are clearly visible in the reconstructed data with no noticeable artifacts. On the magnified images (Figure 5b–e), it is possible to see the smallest features with more details. Another noticeable large scale feature is long‐range phase shift variation over the whole sample with a horizontal gradient, this is the structure of the holder on which the sample was fixed and that was in the beam path.

**Figure 6:**
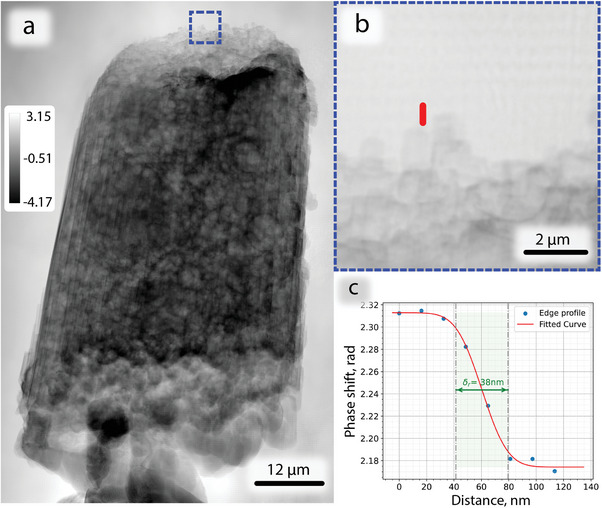
Unwrapped reconstructed object phase from the 6 beam MBP measurement on the Ni/Al_2_O_3_ catalyst sample at 13 keV; a) overall image; b) magnified region; c) line edge profile estimating resolution; The color bars represent phase shift in radians. The pixel size in reconstruction is 16 nm.

A second potential application area of MBP is in the study of complex composite samples with less organized or more random structural features. These may be represented by energy materials (e.g., batteries, fuel cells) or solid catalyst samples for industrial chemistry applications. These are often composite materials, while entire samples can even exceed mm–cm scale. In this context, MBP is particularly interesting as it enables larger fields of view which may be more representative of the parent sample. This is in principle performed without compromising on resolution. Here, a sample of hierarchically porous Ni/Al_2_O_3_ catalyst was prepared as a cylinder of ≈45 µm diameter. Preparation was performed by a focused ion beam (FIB) and the sample was placed on a tomography pin, as described in previous work.^[^ ^30^ ^]^ The sample was designed for 6 beam arrangement 2 × 3 (*H* × *V*) with a corresponding FOV of 65 µm×95 µm. A single projection showing the reconstructed phase image is depicted in **Figure** **6**a. The total phase shift in the object exceeded 2π, and thus the reconstructed image initially contained phase wraps. These were unwrapped during post‐processing using the unwrap function from the scikit package.^[^ ^31^ ^]^ The final reconstruction clearly indicates the presence of the expected complex interior pore network. This was thoroughly characterized in previous ptychographic X‐ray computed tomography (PXCT) studies and consists of a combination of mesopore (2‐20  nm diameter, not resolved) and macropore (>50 nm diameter, resolved) features, with the latter extending up to 2.5 µm.^[^ ^30^ ^]^ Based on the line edge profile in Figure 6b,c the achieved spatial resolution was estimated to be 38 nm. These results are directly comparable to previous PXCT measurements, in which a 15 µm diameter sample with broadly similar structural features was measured with around 56 nm 3D resolution.^[^ ^30^ ^]^ These results indicate that MBP may in principle be extended to long‐range tomographic measurements of large samples, which would greatly exceed the feasible FOV of conventional single beam ptychography due to time constraints.

### 2.3 Multibeam Ptychography at 20 keV: Six Beams

To examine the possibility of performing MBP at even higher energies, additional experiments with 20 keV irradiation were performed at the ESRF‐EBS beamline ID13. It should be noted that despite the high spectral brightness of the upgraded source, the experiment suffered from low photon statistics; at that energy only the Maxipix GaAs detector without integration mode had sufficient quantum efficiency, limiting the total intensity per pixel to 12518 counts. Furthermore, the control software was not able to provide multiple exposures per point scanning regime. Consequently, the recorded intensity per beam during MBP experiments could not reach the same level as with a single beam, which negatively affected the achieved resolution due to the lower signal‐to‐noise ratio (SNR). Because of this, the number of beams was limited to 6, which showed sufficient quality in the reconstructed object. The following examples, therefore, indicate the successful application of high‐energy MBP. Still, the quality of the results under‐represent the potential performance of MBP in future high‐energy experiments with a more appropriate detector such as Timepix4.^[^ ^32^ ^]^

**Figure 7:**
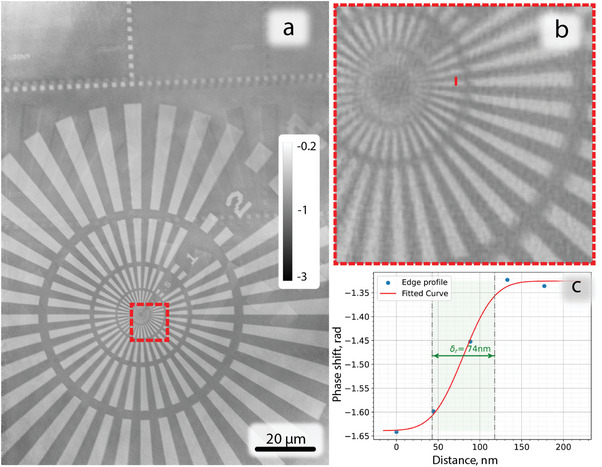
Reconstructed object phase from the 6 beam measurement of the Siemens star test pattern at 20 keV; a) overall image; b) magnified central region; c) line edge profile estimating resolution. The pixel size in reconstruction is 44 nm.

A Siemens star resolution test chart sample was again taken as an initial measurement at 20 keV. The reconstructed image with 6 beams (2 x 3 arranged HxV) is depicted in **Figure** **7**. The estimated resolution according to a line edge profile was 74 nm. This coincides with the fact that the smallest lines and spaces (50 nm) in the center of the test pattern can not be resolved, while the second smallest features of 100 nm were clearly resolved. The image has artifacts in the top region originating from the lack of high diversity of features, leading to reduced quality of the reconstruction.

**Figure 8:**
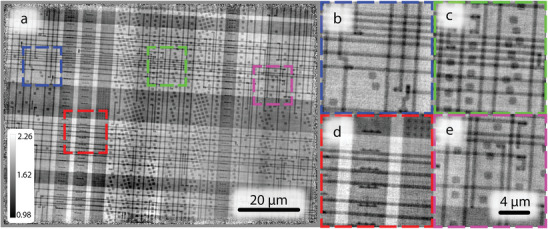
Reconstructed object phase from the 6 beam measurements of the microchip at 20 keV; a) overall image; (b–e) magnified regions indicating small features; The color bar represents phase shift in radians. The pixel size in reconstruction is 44 nm.

**Figure 9:**
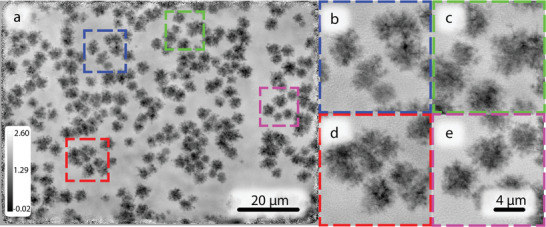
Reconstructed object phase from 6 beam measurement of the gold nano‐crystal cluster on *Si*_3_ *N*_4_ membrane at 20 keV; a) overall image; b–e) magnified regions indicating small features; The color bar represents phase shift in radians. The pixel size in reconstruction is 44 nm.

As with previous experiments at 13 keV, additional samples were chosen with increased structural complexity. First, a microchip with regularly spaced highly diverse features was again measured. The reconstructed phase shift of the microchip sample is depicted in **Figure** **8**. At 20 keV irradiation, the microchip sample has a smaller cross‐section (of radiation–matter interaction), resulting in comparably weaker scattering than at 13 keV. In combination with the reduced photon statistics discussed previously, this led to a lower resolution compared to the 13 keV experiment. Despite this, the image had no noticeable reconstruction artifacts, while different length‐scale sample features were robustly reconstructed. The major difference with the 13 keV experiment is that there was no Al holder in the beam, thus there are no long‐range features in the background of the reconstructed object and all visible structures are a result of the internal components of the microchip. As a final test sample, gold nano‐crystal clusters (**Figure** **9**) were prepared on a SiN membrane with sizes ranging between 1 and 6 µm in size. These represent dispersed sparse objects which are highly scattering and are therefore are interesting test object for MBP since they are non‐contiguous objects on otherwise featureless background. In summary despite reduced photon statistics, MBP is shown to be robust even at the high photon energy of 20 keV.

## 3 Conclusion

Compared to contemporary imaging methods with visible light, X‐rays, or electrons, hard X‐ray ptychography in 2D or 3D offers significant advantages in the study of relatively large objects at the nanoscale. Despite this, a core criticism of ptychography and scanning probe methods more generally, is that they tend to be slow techniques due to the need to scan multiple positions.^[^ ^33^ ^]^ By demonstrating the successful application of MBP with up to 12 beams and at energies of 13 and 20 keV, we present a pathway to extend the use of ptychography to larger samples or more rapid acquisition rates, depending on experimental needs. Crucially, the presented MBP reconstructions achieved the same spatial resolutions as conventional single beam ptychography reconstructions using similar experimental parameters^[^ ^17^ ^]^ (see Supporting Information I). It should be noted that the size and overall design of the conventional test pattern—Siemens star, which was used for resolution comparison, is not ideally suited for assessment of the performance of MBP. This is evident from Figure 4a, wherein the total imaged area is much larger than the high‐resolution zone at the center of the Siemens star, which is normally used to assess the resolution.

Electron microscopy is often regarded as a universal imaging tool in chemistry, physics, and materials science. While it can provide remarkable single‐digit  nm resolution, or even single atoms using electron ptychography, the major drawback comes from sample size limitations. This in principle presents a fundamental physical constraint that cannot be overcome, necessitating the use of hard X‐rays for measuring extended samples. In the case of MBP, it is now proven that it can perform non‐destructive imaging of extended samples with lateral size exceeding the travel range of typical scanning stages and with resolution on the same level as single beam ptychography.

The need to measure extended samples at high resolution is most urgent in the study of functional materials and nanodevices, where invasive subsampling may lead to unrepresentative small volumes. In fields such as energy materials, catalysis, and microelectronics, for example, the ability to measure samples of 100 µm or greater with resolutions of 20 nm or below will open unprecedented possibilities for the accurate characterization of nanoscale structure. In addition to simply measuring larger samples, the use of MBP may in particular enable rapid scans of smaller samples, facilitating the use of in situ methods to image transient processes with greater accuracy (i.e., better time resolution).^[^ ^8^, ^30^, ^34^ ^]^ A further application is in high throughput imaging of samples with large structural variations, such as in heterogeneous catalysis^[^ ^35^ ^]^ in which there can be high variation between individual samples.^[^ ^36^ ^]^ All of the above represent current challenges in X‐ray imaging, all of which may be overcome by MBP as demonstrated here. In particular, the extension of MBP to tomographic regimes would be particularly attractive for such samples due to their complex 3D architecture. However, tomographic experiments will necessitate the use of high‐energy X‐rays, to minimize both beam attenuation and extensive phase artifacts such as wraps or vortices in larger samples.^[^ ^12^ ^]^ Therefore successful demonstration of MBP at 20 keV is a significant step toward future MBP tomography studies of complex solid matter.

The 3D two‐photon printing technique for optics manufacturing applied here has now reached the performance level to deliver high aspect ratio structures with remarkable precision. Here optics were manufactured with an aspect ratio exceeding 100:1 (single lens tower). This is especially important for MBP at higher energies, as it allows maintaining sufficient focusing power and provides sufficient quality of phase coding plates, which is crucial for the successful separation of diffraction signals from different beams. Further optics development for MBP will eventually move toward reducing single‐lens aperture sizes while maintaining sufficient NA, which is dictated by the demands of the acquisition speed. The scanning time in MBP is ultimately limited by the total flux per beam, and the scan range required to cover the full distance between neighboring beams. While the synchrotron ultimately defines the total flux per beam, the scanning range can be varied by reducing the distance between neighboring beams. By reducing the lens aperture, it will be possible to vary the scanning range and thus the scanning time significantly. Additionally, prefocusing may be used to match the coherent length of the synchrotron beam and the lens aperture. In this way, it will be possible to image objects of remarkable size while performing very small scans. Already now, the imaged area of several objects described here exceeded the maximum range of the used scanning stage (100 µm). The ability of the 3D two‐photon printing technique to quickly create virtually any design optics constitutes it as the flexible, precise, and reliable tool for manufacturing on‐demand tailored optics for specific needs of particular experiment and SRF.

Currently, a major direction in MBP development is performing it at even higher energies >25 keV. Here the potential speed gains are especially high, considering the presence of fourth‐generation synchrotrons such as MAX IV^[^ ^37^ ^]^ or ESRF‐EBS, along with future projects such as PETRA IV,^[^ ^38^ ^]^ SPRING 8‐II or Diamond II. At such SRFs, the beam coherence fraction at higher energies (>25 keV) will be comparable with what current sources can achieve at <10 keV. This will open the avenue for imaging of macroscopic samples (such as industrial catalysts) which cannot feasibly be measured with lower energies due to high attenuation of the beam. For this, highly efficient focusing lens arrays will be essential, which will match the current development trend of nano‐lithography optics—high NA small aperture lenses. Another aspect here is the exploration of so‐called “pink beam” measurements. Taking into account the capabilities of new sources in producing more temporally coherent beams, MBP may, with careful execution, increase the speed of data acquisition even further by utilizing currently wasted photons of different energy without requiring significant or perhaps even any monochromatization of the beam.

Another direction of development for MBP is utilization at low‐brilliance sources, such as older synchrotrons or bright laboratory sources. The latter are very attractive as they are widely available and, unlike user facilities, have easy access and affordable maintenance costs for a single scientific group. The knowledge and developments acquired at SRFs can in principle be almost directly applied to implement MBP for high‐resolution quantitative 3D imaging in the laboratory, which opens up new avenues in many fields of science and industry and also significantly reduces the costs of measurements.

In summary, we demonstrate MBP as a high‐value and high‐performance method to image extended samples at the nanoscale. This was achieved due to spectacular progress in 3D lithography which allowed us to manufacture precise and highly efficient tailored optics with integrated coding phase plates. In comparison to conventional hard X‐ray ptychography, which currently offers the highest possible spatial resolution of known X‐ray methods, MBP improves either field of view or scan speed with no compromise on spatial resolution. Due to the broad application fields of hard X‐ray nano‐imaging, and with the efficient preparation of suitable optics, we anticipate that MBP may in principle supersede the use of single beam ptychography for the study of large complex or composite samples.

## 4 Experimental Section

### Lens Array Design and Manufacturing

The lens array was designed in accordance with the following requirements: 1) each individual lens had to be illuminated with a coherent beam, 2) lens spacing had to satisfy cloaking condition – (Equation 6, Supporting Information II), 3) lens focusing power had to be sufficient to make focused beam size to satisfy CDI oversampling criteria for used beamline operation conditions.

**Figure 10:**
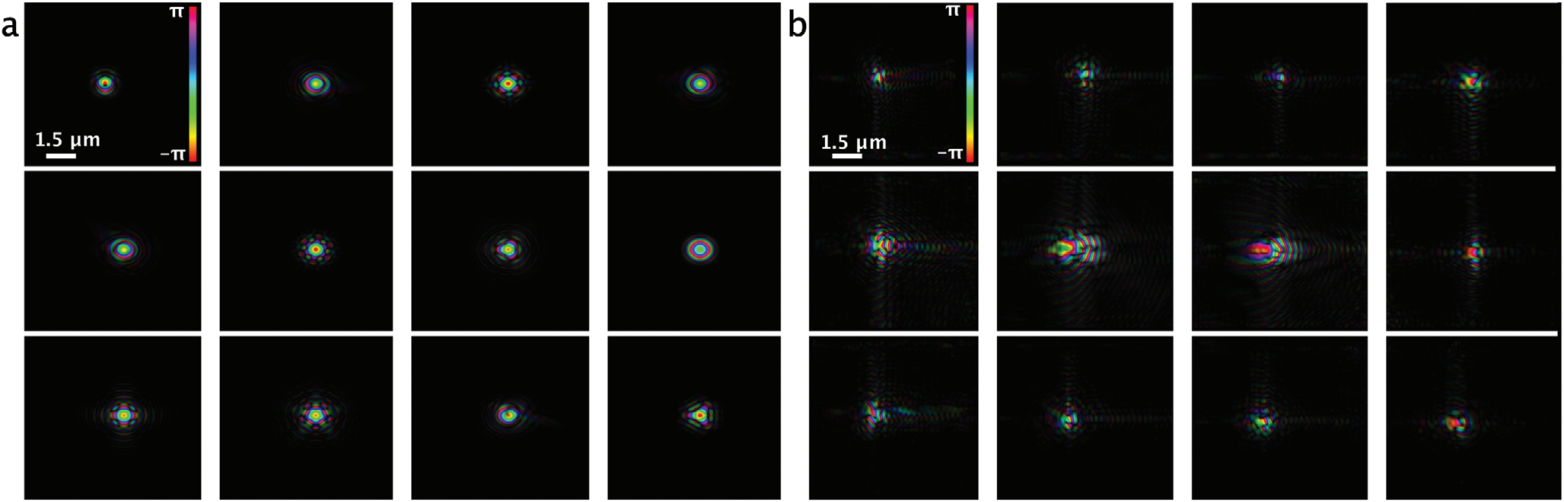
The comparison between the simulated probe and the reconstructed probe from the experiment at 1.5 mm defocus distance at 13 keV; a) the simulated complex wave‐field cross‐section with color‐coded phase, each probe corresponds to each lens tower in the lens array; b) the reconstructed complex wave‐field cross‐section with color‐coded phase. The phase ranges from −π to π.

For robust reconstruction, each beam might be uniquely coded in phase and/or amplitude to help separate superposed scattering signals at the detector. The criterion for phase plate design was that the amplitude on the defocus plane was tightly distributed, and the phase was distinguishable from probe to probe. Therefore, three‐phase plate modals (the cake pieces, pyramid, and vortex layers) were designed. Before manufacturing, the simulation was performed to check the probe difference based on the proper probe size on the sample plane. **Figure** **10**a, shows the probe amplitude with color‐coded phase at 1.5 mm defocus distance from the focal plane. The height of the phase plate designed was on the refractive index of IP‐S materials and the maximum phase shift that the phase plate can bring was approximately π. For the design of the phase plate at 13 keV, the highest structure was about 23 µm. For the design of the phase plate at 20 keV, the highest structure was about 45 µm.

The experimentally retrieved probes are depicted in Figure 10b. The discrepancy was caused by two reasons: the wave fields being very sensitive from the propagation distance down to sub‐ mm which makes it very difficult to find the exact defocus plane as in the experiment; wave fields are also affected by the the lens aberrations. Although it could be difficult to compare the designed and the real experiment probes directly, it does not hinder the purpose of the phase plate – to make probes different.

Lens arrays were fabricated using a commercial 3D laser printing setup (Nanoscribe, Photonics Professional GT) and a commercial photoresist system (Nanoscribe, IP‐S) based on acrylates. A 25X NA1.4 microscope objective lens that provides smooth surfaces when printing micro‐optical components was used. The resulting lateral (axial) voxel size was ≈400 nm (2300 nm). The structures were printed directly on SiN membranes (Norcada NX10100D). Before printing, the membranes were cleaned in a plasma oven and subsequently silanized for better adhesion. All structures were printed with a slicing distance of 300 nm, hatching distance of 200 nm, focus‐scanning speed of 75 mm s^–1^, and laser power of 25 mW (measured at the entrance pupil of the microscope objective lens). Since the focal length of the central lens towers was different, the probe size at the sample plane was different as well. To correct for this in the second manufacturing step, the curvature of the two central lens towers was corrected to match the focal length of the others. A second lens array for 20 keV was manufactured in a similar fashion, but containing 40 lenses in a tower instead of 30 to compensate for the higher incident beam energy. An important parameter in lens array manufacturing was the position of each individual lens with respect to the others over the whole height of the structure (see Figure 3a). Since the structures had an aspect ratio exceeding 100:1, 200 µrad precision with respect to the X‐ray beam was essential for proper alignment to maintain sufficient focusing performance. This constrained the position difference between the first and last lenses in each row with respect to the optical axis to 0.6 µm, which was successfully achieved. To ensure precision printing of the structure of the integrated phase plate, they were characterized during the manufacturing process using in situ QPI technique.^[^ ^28^ ^]^ It reconstructed the phase distribution of the structure from the wide‐field intensity image stack. The images were acquired after printing but before development in the same volume of photoresist using LED with a wavelength of 630 nm. The reconstructed phase distribution corresponded to the amount of printed material and was proportional to the product of the refractive index difference between unpolymerized and polymerized photoresists and the height of the printed structure.

### Ptychographic Experiment at P06 of PETRA III

Partial results of this paper were taken from the nanoprobe end‐station of beamline P06 of PETRA III at DESY in Hamburg, Germany. A 30‐lens array was used to create multiple probes by focusing X‐rays, lenses were arranged in a grid with a spacing of 30 µm. Based on experimentally proved optical constants,^[^ ^22^ ^]^ the calculated photon efficiency of the array was about 86%. The beam was monochromatized with a double crystal Si 111 monochromator. The sample was placed ≈70 mm downstream from the lens array. In front of the sample, a custom‐built pinhole array was situated to reduce background scattering on inhomogeneities and surface roughness of the compound lenses. The detector‐sample distance was 3.08 m. The Eiger (2048x2048 pix) was used as the detector with a 75 µm pixel size. The detector was placed in a vacuum to suppress the air scattering. The scanning was performed in a raster pattern with jittering of positions of 20% of the step size. The step size of 350 nm was set according to the smallest probe size from the beam array at the sample position—‐560 nm. The scanning range of 35 µm was chosen to ensure sufficient overlapping between areas scanned by adjacent probes. The dwell time for the 12 beams experiment was chosen according to the detector saturation, namely 0.1s per scan point. For characterization of individual beams, several measurements were performed with lens tower combinations 2 x 2 and 1 x 1, isolating them with custom pinholes. The step size ranged from 350 to 750 nm and the dwell time ranged from 0.25 to 0.5 s per scan point correspondingly.

### Ptychographic Experiment at ID13 of ESRF

Other results of this paper were taken from the third end‐station of the ID13 beamline of the ESRF‐EBS in Grenoble, France. The 40‐lens array was used to create multiple probes by focusing X‐rays at 20 keV with a design similar to the one used for 13 keV experiment. Based on experimentally proved optical constants,^[^ ^22^ ^]^ the calculated photon efficiency of the array was about 93%. The sample was placed ≈108 mm downstream of the lens array. The detector‐sample distance was 5.03 m. The scan pattern was Fermat spiral mode.^[^ ^39^ ^]^ The position precision was ≈100 nm. The MAXIPIX (516x516 pix) was used as the detector with 55 µm pixel size and placed in the air. The detector was operated in single‐exposure mode with a maximum photon count of 12518. The dwell time for for six beams experiment was chosen according to the detector saturation, namely 0.1 s per scan point. For characterization of single beams, the step size was 500 nm and the dwell time was 0.2 s per scan point. Therefore, the resolution and reconstruction were limited by the SNR and position accuracy. The scanning range of 35 µm was chosen to ensure sufficient overlapping between areas scanned by neighboring probes.

### Reconstruction Approach

For reconstructions two software packages were used, internally built Ptycho and open‐source PtyPy.^[^ ^40^ ^]^ Before multibeam reconstruction was performed, each probe was characterized, created by a single lens stack and retrieved the probe from single‐beam ptychographic reconstruction with the standard ePIE algorithm^[^ ^41^ ^]^ for 1000 iterations. For the Siemens star reconstruction results from P06, the recorded far‐field diffraction patterns were cropped to 512 x 512 pixels centered around the beam axis. The pixel size in the reconstruction was 8.1 nm. The image was reconstructed using 6000 iterations of the ePIE algorithm with the object update strength α = 0.1 and the illumination update strength β = 1.0, followed by 8000 iterations with the stronger object update strength α = 1.0 and the weaker illumination update strength β = 0.1 to further optimize object reconstruction.

For the other reconstruction results from P06, the recorded far‐field diffraction patterns were cropped to 256 x 256 pixels centered around the beam axis. The pixel size in the reconstruction was 16.2 nm. The achieved CDI oversampling rate was 2. Every reconstruction consisted of 2500 iterations of the difference map (DM) algorithm^[^ ^42^ ^]^ followed by 7500 iterations of the maximum‐likelihood algorithm.^[^ ^43^ ^]^

For the reconstruction results from ID13, four probe modes were used for the 6 beams reconstruction to account for some fast beam oscillations. The recorded far‐field diffraction patterns were cropped to 128 x 128 pixels centered around the beam axis. The pixel size in the reconstruction was 44.3 nm. Because this experiment was performed at a higher energy, the size of the probes illuminating the sample increased. The reconstruction scheme was changed by enlarging the probe field of view with a 2 x 2 upsampling.^[^ ^44^, ^45^ ^]^ The resulting CDI oversampling rate was 2.5. Every reconstruction consisted of 1000 iterations of the difference map (DM) algorithm followed by 9000 iterations of the maximum‐likelihood algorithm.

In all reconstructions, to compensate for positioning errors in the scanning stage, the scan positions were numerically refined every 50 iterations to improve the reconstruction.^[^ ^46^, ^47^ ^]^

## Conflict of Interest

The authors declare no conflict of interest.

## Supporting information

Supporting Information

Supporting Information

## Data Availability

The data that support the findings of this study are available from the corresponding author upon reasonable request.
