# Supplementary material for: X‐Ray Multibeam Ptychography at up to 20 keV: Nano‐Lithography Enhances X‐Ray Nano‐Imaging
Source: Adv Sci (Weinh). 2024 Jun 23;11(30):2310075. doi: 10.1002/advs.202310075 (PMC11321614; doi:10.1002/advs.202310075)
Supplement: Supplementary file 1 — Supporting Information [file ADVS-11-2310075-s001.pdf]

ADVANCED SCIENCE
Open Access

Supporting Information

for *Adv. Sci.*, DOI 10.1002/advs.202310075

X-Ray Multibeam Ptychography at up to 20 keV: Nano-Lithography Enhances X-Ray Nano-Imaging

*Tang Li, Maik Kahnt, Thomas L. Sheppard, Runqing Yang, Ken V. Falch, Roman Zvagelsky, Pablo Villanueva-Perez, Martin Wegener and Mikhail Lyubomirskiy**

Supplementary Information I - X-ray multibeam ptychography at up to 20 keV: nano-lithography enhances X-ray nano-imaging

Tang Li [1], Maik Kahnt [2], Thomas L. Sheppard [3,#], Runqing Yang [4], Ken Vidar Falch [1], Roman Zvagelsky [5], Pablo Villanueva-Perez [4], Martin Wegener [5], and Mikhail Lyubomirskiy [1,*]

[1] Centre for X-ray and Nano Science CXNS, Deutsches Elektronen-Synchrotron DESY, Notkestr. 85, 22607 Hamburg, Germany

[2] MAX IV Laboratory, Lund University, Box 118, 221 00, Lund, Sweden

[3] Karlsruhe Institute of Technology, Institute for Chemical Technology and Polymer Chemistry, Engesserstr. 20, 76131 Karlsruhe

[4] Division of Synchrotron Radiation Research and NanoLund, Department of Physics, Lund University, Lund, 22100, Sweden

[5]Karlsruher Institut für Technologie, Institut für Angewandte Physik, Wolfgang-Gaede-Straße 1, D-76131, Karlsruhe, Germany

* mikhail.lyubomirskiy@desy.de

# Current address: Leipzig University, Institute of Chemical Technology, Linnéstr. 3, 04103 Leipzig, Germany

Here, we compare the resolution between the single beam ptychographic reconstruction and the MBP reconstruction. For fair comparison, the data-sets should have similar photon statistics. The characterization of individual lens towers was performed with 0.5 s exposure per scan point. During 12 beam experiment the exposure time was reduced to 0.1 s to avoid detector saturation. In order to compare resolution in two scans with the same photon statics per irradiating beam, single beam data was normalized, rounding error was less than 1 count per pixel. Despite the different Poisson noise in two scans (in favor for single beam scan) we have comparable resolution between single-beam ptychographic reconstruction and MBP reconstruction, see S 1.

S 1 shows the reconstruction phase from the scan taken for the single lens tower characterisation with normarlized statistics per irradiating beam and indicates a resolution of 34 nm.

S 2 shows the amplitude of the reconstructed 12 beams at 13 keV

S 3 shows a typical convergence curve of the reconstruction. When the error between the iterations is kept constant, we stop the iteration.

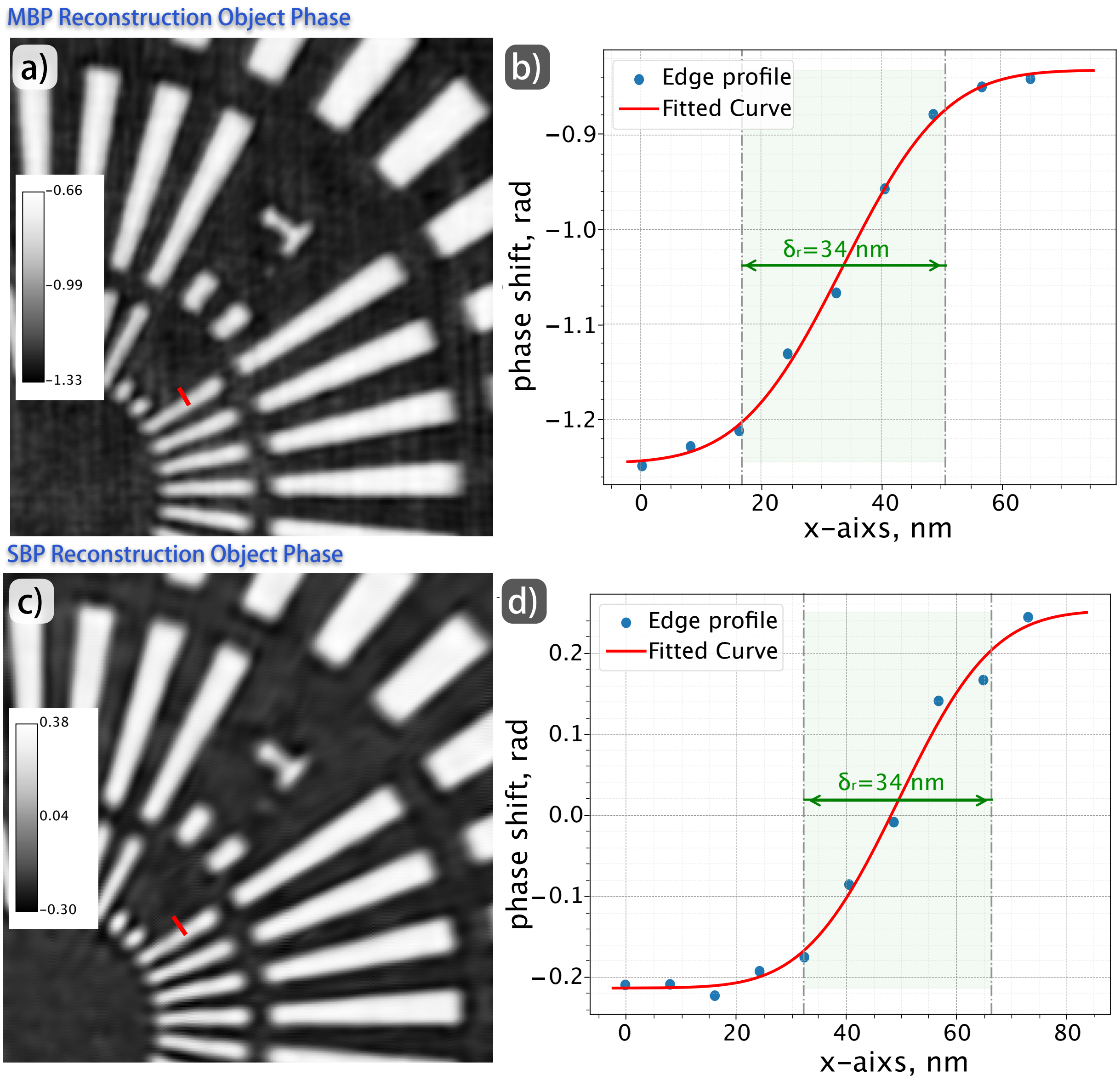

S 1. (a) Reconstructed object phase from the 12 beam measurement at 13 keV of the Siemens star zoomed in the central region, red line indicated the position for the line profile;(b) line profile of the edge estimating resolution;(c) Reconstructed object phase from the single beam measurement of the Siemens star zoomed in the central region, red line indicated the position for the line profile; (d) line profile of the edge estimating resolution. The pixel size in reconstruction is 8 nm.

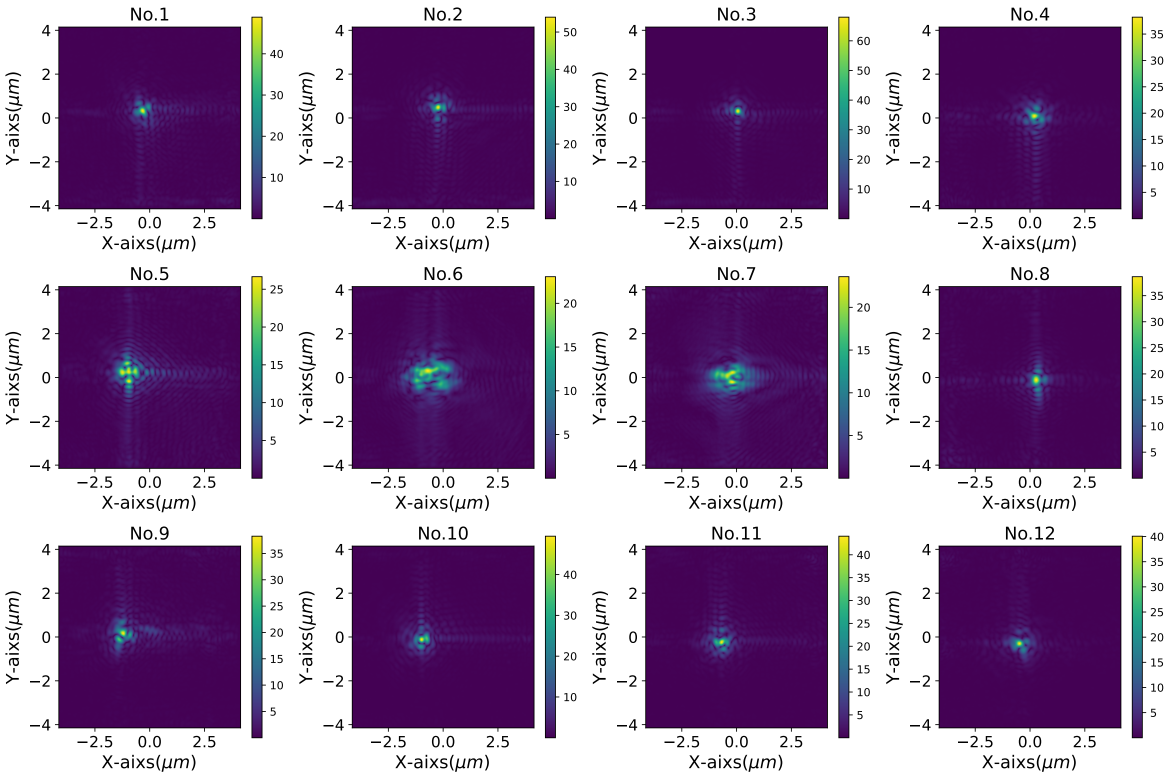

S 2. Reconstructed 12 beam amplitude distribution at 13 keV

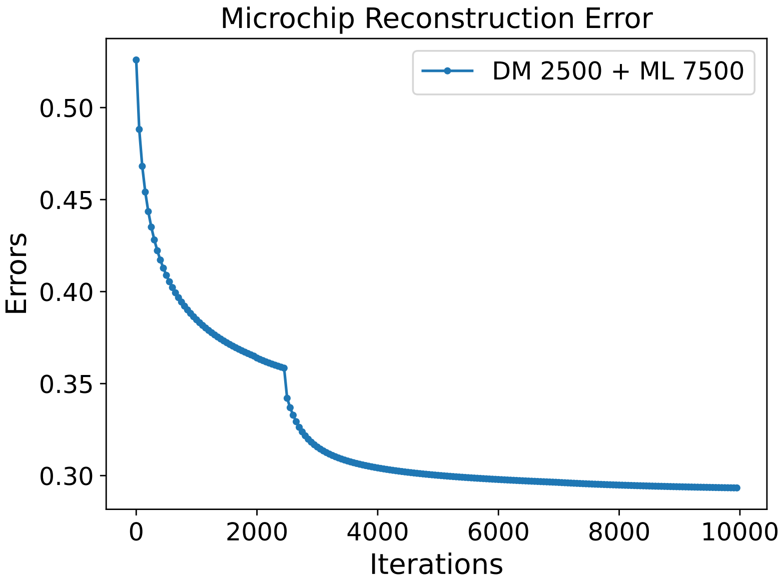

S 3. The convergence curve of microchip reconstruction with 2500 iterations DM and 7500 iterations ML
