# Supplementary material for: X‐Ray Multibeam Ptychography at up to 20 keV: Nano‐Lithography Enhances X‐Ray Nano‐Imaging
Source: Adv Sci (Weinh). 2024 Jun 23;11(30):2310075. doi: 10.1002/advs.202310075 (PMC11321614; doi:10.1002/advs.202310075)
Supplement: Supplementary file 2 — Supporting Information [file ADVS-11-2310075-s002.pdf]

ADVANCED SCIENCE
Open Access

Supporting Information

for *Adv. Sci.*, DOI 10.1002/advs.202310075

X-Ray Multibeam Ptychography at up to 20 keV: Nano-Lithography Enhances X-Ray Nano-Imaging

*Tang Li, Maik Kahnt, Thomas L. Sheppard, Runqing Yang, Ken V. Falch, Roman Zvagelsky, Pablo Villanueva-Perez, Martin Wegener and Mikhail Lyubomirskiy**

Supplementary Information II - X-ray multibeam ptychography at up to 20 keV: nano-lithography enhances X-ray nano-imaging

Tang Li [1], Maik Kahnt [2], Thomas L. Sheppard [3,#], Runqing Yang [4], Ken Vidar Falch [1], Roman Zvagelsky [5], Pablo Villanueva-Perez [4], Martin Wegener [5], and Mikhail Lyubomirskiy [1,*]

[1] Centre for X-ray and Nano Science CXNS, Deutsches Elektronen-Synchrotron DESY, Notkestr. 85, 22607 Hamburg, Germany
[2] MAX IV Laboratory, Lund University, Box 118, 221 00, Lund, Sweden
[3] Karlsruhe Institute of Technology, Institute for Chemical Technology and Polymer Chemistry, Engesserstr. 20, 76131 Karlsruhe
[4] Division of Synchrotron Radiation Research and NanoLund, Department of Physics, Lund University, Lund, 22100, Sweden
[5]Karlsruher Institut für Technologie, Institut für Angewandte Physik, Wolfgang-Gaede-Straße 1, D-76131, Karlsruhe, Germany
* mikhail.lyubomirskiy@desy.de
# Current address: Leipzig University, Institute of Chemical Technology, Linnéstr. 3, 04103 Leipzig, Germany

For initial reconstructions we have adapted multiplex approach from Batey et. al[**1**] to reconstruct spatially separated beams instead of different wavelengths. Here we briefly explain the difference between conventional single beam ptychography algorithms, such as ePIE, difference map (DM) and maximum likelihood (ML)[**2, 3, 4**] and its multibeam counterpart.

A single beam Ptychographic model describes the scattered wave-field at the detector plane as multiplication of the object and the probe at j scan position relative to the sample plane:

(1) $$\psi_j(\mathbf{r}) = O(\mathbf{r}) \cdot P(\mathbf{r} - \mathbf{r}_j)$$

where $\mathbf{r} = (x, y)$ is a coordinate in the plane perpendicular to the propagation direction. For the multibeam case, m probes with offsets $\mathbf{r}_m$ illuminate different positions of the object simultaneously. For each probe m_i, the exit wavefiled can be described as:

(2) $$\psi_{j,m_i}(\mathbf{r}) = O(\mathbf{r}) \cdot P(\mathbf{r} - \mathbf{r}_j - \mathbf{r}_{m_i})$$

where $\mathbf{r}_{m_i} = (n_x \cdot S_m, n_y \cdot S_m)$, S_m is the beam spacing, n_x and n_y is integer. n_x represents the probe index in the horizontal direction and n_y represents the probe index in the vertical direction. For example, if the lens array is 4×3 ($H \times V$), n_x is in the range (0,3) and n_y is in the range (0,2). The propagated wavefront from each beam at the detector plane can be described in the form of Fourier transform:

$$\Psi_{j,m_i}(\mathbf{q}) = \mathcal{F}(\psi_{j,m_i}(\mathbf{r})) \tag{3}$$

Then, the intensity Is recorded at the detector in the case of one probe described as:

$$Is_j(\mathbf{q}) = |\Psi_{j,m_i}(\mathbf{q})|^2, \tag{4}$$

and, in case of multiple incoherent to each other beams, they add up incoherently and intensity I can be represented as:

$$I_j(\mathbf{q}) = \sum_{i=1}^{m} |\Psi_j, m_i(\mathbf{q})|^2. \tag{5}$$

The way to suppress the coherence effects is to choose the beam spacing distance S_m which satisfy alias cloaking condition[**5, 6**]:

$$S_m = n_t \frac{d\lambda}{p}, \tag{6}$$

where p is a detector pixel size, λ is X-rays wavelength, d is a sample-detector distance, and n_t is an integer. The optimum n_t is determined by the coherence length of the forward beam.

1. ePIE multibeam model

To reconstruct the object and probe, we compare the modelled intensities to the measurements using a quadratic error function:

$$\mathcal{L} = \sum_j \sum_{\mathbf{q}} (\sqrt{I_j(\mathbf{q})} - \sqrt{n_j(\mathbf{q})})^2 \tag{7}$$

This error function quantifies the difference between the modelled diffraction patterns $I_j(\mathbf{q})$ and the measured diffraction patterns $n_j(\mathbf{q})$. The object and probe with the minimum error solve the phase problem owing to the redundancy of the data.To find the minimum error, we calculate the gradient by differentiating $\mathcal{L}$ with respect to the object and probe, using Wirtinger derivatives. The gradient with respect to the object and to the probe can be formed as follows:

$$\frac{\partial \mathcal{L}}{\partial O_{\mathbf{r}}} = \sum_j \sum_{m_i=1}^{m} P(\mathbf{r} - \mathbf{r}_j - \mathbf{r}_{m_i}) \cdot \phi^*_{j,m_i}(\mathbf{r}), \tag{8}$$

$$\frac{\partial \mathcal{L}}{\partial P_{\mathbf{r}}} = \sum_j \sum_{m_i=1}^{m} O(\mathbf{r} + \mathbf{r}_j + \mathbf{r}_{m_i}) \cdot \phi^*_{j,m_i}(\mathbf{r} + \mathbf{r}_j + \mathbf{r}_{m_i}). \tag{9}$$

In ePIE algorithm, we use stochastic gradient descent which splits the gradient calculation and gradient step into subiterations. Rather than computing the full gradient before each step, we choose a random diffraction pattern, compute its gradient, and make a gradient step. We continue by selecting unused patterns in subsequent subiterations, computing their gradients, and updating in these directions. An iteration finishes once every pattern has been used. In each iteration of gradient descent, we calculate the gradient and take one step proportional to the negative of the gradient. The update functions for object and probes in multibeam case can be represented:

$$O_{l+1}(\mathbf{r}) = O_l(\mathbf{r}) + \alpha \cdot \sum_{i=1}^{m} \frac{P^*_{l,m_i}(\mathbf{r}-\mathbf{r}_j-\mathbf{r}_{m_i})}{max|P_l(\mathbf{r}-\mathbf{r}_j-\mathbf{r}_{m_i}|^2)} \cdot -\phi_{j,m_i}(\mathbf{r}) \tag{10}$$

$$P_{l+1,m_i}(\mathbf{r}) = P_{l,m_i}(\mathbf{r}) + \beta \cdot \frac{O^*_l(\mathbf{r}+\mathbf{r}_j+\mathbf{r}_{m_i})}{max|O_l(\mathbf{r}+\mathbf{r}_j+\mathbf{r}_{m_i})|^2} \cdot -\phi_{j,m_i}(\mathbf{r}+\mathbf{r}_j+\mathbf{r}_{m_i}), \tag{11}$$

$$\phi_{j,m_i}(\mathbf{r}) = \mathcal{F}^{-1}((1-\frac{\sqrt{n_j(\mathbf{q})}}{\sqrt{I_j(\mathbf{q})}})\Psi_{j,m_i}(\mathbf{q}) \tag{12}$$

In Eq.(10), the object is updated with the changes from each wavefront ϕ_{m_i}. Here, α is the object update strength and β is the illumination update strength.

2. DM algorithm multibeam model

In difference map (DM) algorithm, the multibeam ptychographic problem Eq.(5) can be re-expressed as two intersecting constraints by introducing the exit waves ψ_{j,m_i} (the "views" on the sample) corresponding to each position of each probe on the sample. The Fourier constraint expresses compliance to the measured intensities and the overlap constraint states that each view can be factorized as a set of probes and an object function, as Eq.(2).

After the reformulation, we can choose a high dimensional Euclidean space to embed the two constraint sets. This search space, in which iterations will take place, is the direct product of the spaces of each individual view. For the multibeam case, a state vector can be rewritten as:

$$\Psi = (\sum_{m_i=1}^{m} \psi_1(\mathbf{r}); \sum_{m_i=1}^{m} \psi_2(\mathbf{r}); ...; \sum_{m_i=1}^{m} \psi_N(\mathbf{r})) \tag{13}$$

This assumption holds when the probes are incoherent to each other. Then the subsequent calculations are dictated by the difference map formalism. The algorithm requires distance-minimizaing projections onto the constraint sets. These operations modify minimally an input state vector ψ to satisfy a given constraint.

2.1. Constraint I: Fourier constraint. The projection associated to the Fourier constraint $\prod_F$ simply consists in applying the Fourier projection, denoted by $\mathbf{p}_F$, on each individual view:

$$\prod_F(\Psi) : \sum_{m_i=1}^{m} \psi_{j,m_i} \to \sum_{m_i=1}^{m} \psi_{j,m_i}^F = \mathbf{p}_F(\sum_{m_i=1}^{m} \psi_{j,m_i}) \tag{14}$$

Given an estimate $\sum_{m_i=1}^{m} \psi_{j,m_i}$ of the jth view, $\mathbf{p}_F(\sum_{m_i=1}^{m} \psi_{j,m_i})$ is obtained by replacing the magnitudes of the Fourier transform by $\sqrt{n_j}$ while keeping the original phases.

2.2. Constraint II: Fourier constraint. The overlap projection can be computed from the minimization of the distance $\parallel \Psi - \Psi^0 \parallel^2$,subject to the constraint Eq.(2). The calculation entails to finding $\hat{O}$ and $\hat{P}$ that minimise

$$\parallel \Psi - \Psi^O \parallel^2 = \sum_j \sum_{\mathbf{r}} \sum_{m_i=1}^{m} \left| \psi_{j,m_i}(\mathbf{r}) - \hat{P}(\mathbf{r} - \mathbf{r}_j - \mathbf{r}_{m_i})\hat{O}(\mathbf{r}) \right|^2 . \tag{15}$$

The associated projection is

$$\prod_O(\Psi) : \psi_{j,m_i} \to \psi_{j,m_i}^O(\mathbf{r}) = \hat{P}(\mathbf{r} - \mathbf{r}_j - \mathbf{r}_{m_i})\hat{O}(\mathbf{r}). \tag{16}$$

Minimization of Eq.(15) is carried numerically using such as conjugate gradients minimization. Here, the derivative of $\parallel \Psi - \Psi^O \parallel^2$ setting to zero with respect to $\hat{P}$ and $\hat{O}$ gives the solution as a system of equations

$$\hat{O}(\mathbf{r}) = \frac{\sum_{j,m_i} \hat{P}^*(\mathbf{r} - \mathbf{r}_j - \mathbf{r}_{m_i})\psi_{j,m_i}(\mathbf{r})}{\sum_{j,m_i} \left| \hat{P}(\mathbf{r} - \mathbf{r}_j - \mathbf{r}_{m_i}) \right|^2}, \tag{17}$$

$$\hat{P}(\mathbf{r}) = \frac{\sum_{j,m_i} \hat{O}^*(\mathbf{r} + \mathbf{r}_j + \mathbf{r}_{m_i})\psi_{j,m_i}(\mathbf{r} + \mathbf{r}_j + \mathbf{r}_{m_i})}{\sum_{j,m_i} \left| \hat{O}(\mathbf{r} + \mathbf{r}_j + \mathbf{r}_{m_i}) \right|^2}. \tag{18}$$

Both Eq.(17) and Eq.(18) need to be simultaneously solved.

2.3. Error Metric. The reconstruction is implemented with the above projections. The iterative procedure takes the following form,

$$\Psi_{n+1} = \Psi_n + \prod_F[2\prod_O(\Psi_n) - \Psi_n] - \prod_O(\Psi_n) \tag{19}$$

Iteration of Eq.(19) is carried until a fixed point is reached, i.e. $\Psi_{n+1} = \Psi_n$. Convergence is calculated with the DM error,

$$\epsilon_{n+1} = \parallel \Psi_{n+1} - \Psi_n \parallel \tag{20}$$

Eq.(20) indicates the distance between the constraint sets near Ψ_n.

Another error metric "R-factor" can modify it based on multibeam ptychographic model, Eq.(21) then takes the form

$$R = \frac{\sum_{\mathbf{q},j} \left| \sqrt{n_j(\mathbf{q})} - \left| \sum_{m_i=1}^{m} \mathcal{F}(P(\mathbf{r} - \mathbf{r}_j - \mathbf{r}_{m_i}) O(\mathbf{r}) \right| \right|}{\sum_{\mathbf{q},j} \sqrt{n_j(\mathbf{q})}} \tag{21}$$

3. Maximum Likelihood (ML) multibeam model

As we have already known the forward model of multibeam ptychography which is shown in Eq.(5). For ML reconstruction algorithm, we will focus on reform the probabilistic description of the data acquisition based on multibeam model. The reconstruction problem is to find a series of pairs $(P(\mathbf{r} - \mathbf{r}_{m_i})_{m_i=(1,\ldots,m)}, O(\mathbf{r}))$ that satisfies Eq.(3). Here, we first consider the counting statistics in ideal case which follows the Poisson distribution. The probalibility of measuring $n_{\mathbf{q}}$ photons given a sum of $P(\mathbf{r} - \mathbf{r}_{m_i})$ and $O(\mathbf{r})$ is

$$p(n_{j,\mathbf{q}} | \sum_{m_i=1}^{m} P(\mathbf{r} - \mathbf{r}_{m_i}), O(\mathbf{r})) = \frac{(I_j(\mathbf{q}))^{n_j(\mathbf{q})}}{n_j(\mathbf{q})!} e^{-I_j(\mathbf{q})} \tag{22}$$

where, $I_j(\mathbf{q})$ is a function of $\sum_{m_i=1}^{m} P(\mathbf{r} - \mathbf{r_m}), O(\mathbf{r})$ as defined in Eq.(5). The negative log-likelihood function associated with this probability distribution is a function of the observed intensity data $n_j(\mathbf{q})$:

$$\begin{aligned} \mathcal{L} &= -log \prod_j \prod_{\mathbf{q}} p(n_j(\mathbf{q}) | \sum_{m_i=1}^{m} P(\mathbf{r} - \mathbf{r}_{m_i})) \\ &= -\sum_j \sum_{\mathbf{q}} \mathrm{w_j}(\mathbf{q})[\mathrm{n_j}(\mathbf{q})\log(\mathrm{I_j}(\mathbf{q})) - \mathrm{I_j}(\mathbf{q}) - \log(\mathrm{n_j}(\mathbf{q})!)] \end{aligned} \tag{23}$$

Here, $\mathrm{w_j}(\mathbf{q})$ is the mask. The maximum of the likelihood, which is equal to the minimization of this function, requires the calculation of its gradient for any $P(\mathbf{r} - \mathbf{r}_{m_i}), O(\mathbf{r})$. Because the intensity is a function of the product Eq.(2), the derivatives can be written as

$$\mathrm{g}_{\mathrm{O}_{\mathbf{r}}} = \frac{\partial \mathcal{L}}{\partial \mathrm{O}_{\mathbf{r}}} = \sum_{\mathrm{j}} \sum_{\mathrm{m_i}=1}^{\mathrm{m}} \mathrm{P}(\mathbf{r} - \mathbf{r}_{\mathrm{j}} - \mathbf{r}_{\mathrm{m_i}}) \mathcal{X}_{\mathrm{j}}^{*}(\mathbf{r}) \tag{24}$$

$$\mathrm{g}_{\mathrm{P}_{\mathbf{r}}} = \frac{\partial \mathcal{L}}{\partial \mathrm{P}_{\mathbf{r}}} = \sum_{\mathrm{j}} \sum_{\mathrm{m_i}=1}^{\mathrm{m}} \mathrm{O}(\mathbf{r} + \mathbf{r}_{\mathrm{j}} + \mathbf{r}_{\mathrm{m_i}}) \mathcal{X}_{\mathrm{j}}^{*}(\mathbf{r} + \mathbf{r}_{\mathrm{j}} + \mathbf{r}_{\mathrm{m_i}}) \tag{25}$$

where * denotes the complex conjugation and the auxiliary function $\mathcal{X}_j(\mathbf{r})$ can be expressed in Fourier space:

$$\tilde{\mathcal{X}}_j(\mathbf{q}) = \frac{\partial \mathcal{L}}{\partial I_j(\mathbf{q})} \sum_{m_i=1}^{m} \tilde{\psi}_{j,m_i}(\mathbf{q})$$
(26)
$$= \mathrm{w_j}(\mathbf{q})(1 - \frac{\mathrm{n_j}(\mathbf{q})}{\mathrm{I_j}(\mathbf{q})}) \sum_{\mathrm{m_i=1}}^{\mathrm{m}} \tilde{\psi}_{\mathrm{j,m_i}}(\mathbf{q})$$

If we consider additional sources of error, then the appropriate likelihood function is instead based on a Gaussian distribution, where the spatially dependent measurement uncertainties are $\sum_{m_i=1}^{m} \sigma_{j,m_i}(\mathbf{q})$. The corresponding negative log-likelihood rewrite as the form of a weighted sum of squares:

$$\mathcal{L} = \sum_j \sum_{\mathbf{q}} \frac{\mathrm{w}_{\mathrm{j},\mathbf{q}}}{2\left|\sum_{m_i=1}^{m} \sigma_{j,m_i}(\mathbf{q})\right|^2} (I_j(\mathbf{q}) - n_j(\mathbf{q}))^2 \tag{27}$$

Computing the gradient of $\mathcal{L}$ also leads to terms in the form Eq.(24) and Eq.(25):

$$\tilde{\mathcal{X}}_j(\mathbf{q}) = \mathrm{w_j}(\mathbf{q}) \frac{\mathrm{I_j}(\mathbf{q}) - \mathrm{n_j}(\mathbf{q})}{\sigma_{\mathrm{j}}^2(\mathbf{q})} \tilde{\psi}_{\mathrm{j}}(\mathbf{q}) \tag{28}$$

In particular case only the Poission variance contributes to the measurement uncertainty, one could substitute $\left(\sum_{m_i=1}^{m} \sigma_{j,m_i}(\mathbf{q})\right)^2 = n_j(\mathbf{q})$ to obtain a form which is similar to Eq.(26):

$$\tilde{\mathcal{X}}_j(\mathbf{q}) = \mathrm{w_j}(\mathbf{q})(\frac{\mathrm{I_j}(\mathbf{q})}{\mathrm{n_j}(\mathbf{q})} - 1) \sum_{\mathrm{m_i=1}}^{\mathrm{m}} \tilde{\psi}_{\mathrm{j,m_i}}(\mathbf{q}) \tag{29}$$

This approximation is valid for high photon counts, where the Poisson distribution approaches a Gaussian. Another possible path to approximating the Poisson likelihhod function is to substitute $\sum_{m_i=1}^{m} F_{j,m_i}(\mathbf{q}) = \sqrt{I_j(\mathbf{q})}$ into Eq.(23) and expanding around $F_j(\mathbf{q}) = \sqrt{n_j(\mathbf{q})}$, then the negative log-likelihood can be rewritten as:

$$\mathcal{L} \approx \sum_{m_i=1}^{m} \sum_j \sum_{\mathbf{q}} 2\mathrm{w_j}(\mathbf{q})(\mathrm{F_j}(\mathbf{q}) - \mathrm{n_j}(\mathbf{q}))^2 \tag{30}$$

Based on Eq.(30), the gradient of it can again be written in the form Eq.(24) and Eq.(25) with

$$\tilde{\mathcal{X}}_j(\mathbf{q}) = 2\mathrm{w_j}(\mathbf{q})(1 - \frac{\sqrt{\mathrm{n_j}}(\mathbf{q})}{\sum_{\mathrm{m_i=1}}^{\mathrm{m}} \left|\tilde{\psi}_{\mathrm{j,m_i}}(\mathbf{q})\right|}) \sum_{\mathrm{m_i=1}}^{\mathrm{m}} \tilde{\psi}_{\mathrm{j,m_i}}(\mathbf{q}) \tag{31}$$

The case when a single diffraction pattern appears in Eq.(30) corresponds to conventional multi-beam diffractive imaging. Since the log-likelihood coincides with the metric of the

search space, the gradient has a clear geometric interpretation: its negative points in the direction of the projection of $\psi_{j,m_i}(\mathbf{r})$ onto the set of elements satisfying $\sum_{m_i} |\psi_{j,m_i}(\mathbf{q})| = \sum_{m_i} \mathcal{F}_{j,m_i}(\mathbf{q})$.

Ptychographic reconstruction including measurement statistics amounts to minimizing Eq.(23), Eq.(27) or Eq.(30). In this paper's ML reconstruction, we use photon statistics model based on Gaussian distribution.

References

[1] Darren J. Batey, Daniel Claus, and John M. Rodenburg. Information multiplexing in ptychography. *Ultramicroscopy*, 138:13–21, 2014.

[2] Andrew M. Maiden and John M. Rodenburg. An improved ptychographical phase retrieval algorithm for diffractive imaging. *Ultramicroscopy*, 109(10):1256–1262, 2009.

[3] Pierre Thibault, Martin Dierolf, Oliver Bunk, Andreas Menzel, and Franz Pfeiffer. Probe retrieval in ptychographic coherent diffractive imaging. *Ultramicroscopy*, 109(4):338–343, 2009.

[4] P Thibault and M Guizar-Sicairos. Maximum-likelihood refinement for coherent diffractive imaging. *New Journal of Physics*, 14(6):063004, jun 2012.

[5] Charles Bevis, Robert Karl, Jonathan Reichanadter, Dennis F. Gardner, Christina Porter, Elisabeth Shanblatt, Michael Tanksalvala, Giulia F. Mancini, Henry Kapteyn, Margaret Murnane, and Daniel Adams. Multiple beam ptychography for large field-of-view, high throughput, quantitative phase contrast imaging. *Ultramicroscopy*, 184:164–171, 2018.

[6] Felix Wittwer. *Development and study of refractive phase retrieval and X-ray multibeam ptychography*. Dissertation, University of Hamburg, Hamburg, 2020.
